# Retraction: Icariin enhances intestinal barrier function by inhibiting NF-κB signaling pathways and modulating gut microbiota in a piglet model

**DOI:** 10.1039/d3ra90005c

**Published:** 2023-01-16

**Authors:** Wen Xiong, Haoyue Ma, Zhu Zhang, Meilan Jin, Jian Wang, Yuwei Xu, Zili Wang

**Affiliations:** a College of Animal Science and Technology, Southwest University Chongqing China pansquito@hotmail.com zhuzi625@sina.com meilan0622@swu.edu.cn jane0931@126.com xuyuwei@swu.edu.cn wzl9698@126.com; b College of Pharmaceutical Sciences, Southwest University Chongqing China mhy950708@vip.qq.com

## Abstract

Retraction of ‘Icariin enhances intestinal barrier function by inhibiting NF-κB signaling pathways and modulating gut microbiota in a piglet model’ by Wen Xiong *et al.*, *RSC Adv.*, 2019, **9**, 37947–37956, https://doi.org/10.1039/C9RA07176H.

The Royal Society of Chemistry hereby wholly retracts this *RSC Advances* article due to concerns with the reliability of the data.

The first three bands in the ‘ZO-1’ western blot panel in Fig. 4B are duplicated as the last three bands in the ‘MyD88’ panel in Fig. 5B. The β-actin control panel in Fig. 4B is duplicated in Fig. 5B. In addition, in the ‘Materials and methods’ section, the authors state that GAPDH was used as the reference gene, which does not match with the information presented in Fig. 4B and 5B where β-actin is used as the control.

Furthermore, the western blot panels, ‘ZO-1’ (Fig. 4B) and ‘MyD88’ (Fig. 5B) were previously published in another paper with no authors in common.^1^ There is also considerable text overlap in the ‘Results’ and ‘Materials and methods’ sections with ref. 1.

Given the significance of the concerns about the validity of the data, and the lack of raw data, the findings presented in this article are not reliable.

The authors stated that a third-party testing company conducted the western blot analysis, and the authors directly used the results sent to them by the company in the paper. In previous correspondence, all the authors agreed to voluntarily request the retraction of this manuscript in order to avoid misleading readers.

Signed: Laura Fisher, Executive Editor, *RSC Advances*

Date: 11th January 2023

## Supplementary Material
